# Treatment Response to Gabapentin in Neuropathic Ocular Pain Associated with Dry Eye

**DOI:** 10.3390/jcm9113765

**Published:** 2020-11-22

**Authors:** Hyeon-Jeong Yoon, Jonghwa Kim, Kyung Chul Yoon

**Affiliations:** Department of Ophthalmology, Chonnam National University Medical School and Hospital, 42 Jebong-ro, Dong-gu, Gwangju 61469, Korea; yoonhyeonjeong@hanmail.net (H.-J.Y.); ccaaacc@hanmail.net (J.K.)

**Keywords:** gabapentin, dry eye, neuropathic pain

## Abstract

Purpose: To investigate the response to gabapentin treatment in patients with dry eye (DE) accompanied by features of neuropathic ocular pain (NOP), and to analyze the differences between clinical manifestations of the groups according to treatment response. Methods: We retrospectively reviewed the records of 35 patients with DE accompanied by NOP features and obtained information on their medical history and previous ocular history. The patients underwent clinical examinations of the tear film, ocular surface, and meibomian gland and completed the Ocular Pain Assessment Survey (OPAS). One month after treatment with topical eye drops, add-on of gabapentin treatment was determined according to the Wong–Baker FACES Pain Rating Scale (WBFPS). A reduction of 2 points or more on the WBFPS was considered a positive treatment response. Enrolled patients were divided into three groups according to the treatment response: topical treatment response group (group 1, *n* = 11); gabapentin response group (group 2, *n* = 13); and gabapentin non-response group (group 3, *n* = 11). The medical history, clinical parameters, and OPAS scores were compared between groups. Results: The incidence of systemic comorbidities was higher in group 2 than in other groups. The corneal staining scores were lower in groups 2 and 3 than in group 1. Among the treatment response groups, group 2 showed improvements in OPAS scores of ocular pain severity, pain other than eyes, and quality of life, while group 1 showed improved OPAS scores of ocular pain severity and ocular associated factors. Group 2 exhibited lower scores of pains aggravated by mechanical and chemical stimuli than group 3. Conclusions: Gabapentin could be effective in patients who have systemic comorbidity and less pain evoked by mechanical and chemical stimuli for the treatment of DE patients with NOP, which is refractory to topical treatment.

## 1. Introduction

Dry eye (DE) is a multifactorial disease of the ocular surface characterized by a loss of homeostasis of the tear film and accompanied by ocular symptoms [1]. The prevalence of DE has increased considerably worldwide over the last three decades [2]. Some patients with DE experience severe pain that reduces their quality of life (QoL) with minimal ocular surface signs [1,3]. Their manifestations include a variety of unpleasant spontaneous ocular sensations, such as burning, aching, and photoallodynia [1,3]. A neurobiological mechanism is known to underline the ocular symptoms of DE [3].

The classification of pain is based on the underlying etiology: nociceptive, neuropathic, and mixed [4]. The primary approach to treating severe ocular pain is to target potential nociceptive factors [5]. Topical treatment could be attempted to lower tear osmolarity and reduce inflammation [6]. If the pain cannot be resolved with the primary approach, neuropathic pain could be considered [5,7,8]. In DE, persistent damage to the ocular surface and nerve endings induced by tear film instability and inflammation can cause peripheral neuronal sensitization. Moreover, repeated peripheral nerve injury can lead to central neuronal sensitization [9,10,11,12].

DE and neuropathic pain share several common features, including frequent discordance between the symptoms and signs, abnormal somatosensory testing, accompanying comorbidity, and anatomic nerve injury or somatosensory nerve sensitization [9,13]. Ocular pain symptoms disproportionally outweighing the clinical signs are suggestive of an underlying neuropathic etiology that requires systemic pain management [9,13].

Oral gabapentin is the first-line agent for the treatment of chronic systemic neuropathic pain [14]. Especially, gabapentin could reverse elements of central sensitization in patients with chronic pain [15,16,17]. This agent has been studied as a therapy for ocular pain after refractive surgery and painful DE [7,18,19,20]. However, central nervous system (CNS) depression may occur as a side effect [15], and the therapeutic efficacy of gabapentin for neuropathic ocular pain (NOP) has not been verified. Hence, ophthalmologists are less likely to use gabapentin for treating NOP which is refractory to topical agents. In this study, we aimed to investigate the response to gabapentin treatment in DE patients with NOP features and compare the clinical parameters and ocular pain assessment survey (OPAS) scores between groups according to the treatment response.

## 2. Methods

Ethical approval was obtained from the Chonnam National University Hospital Institutional Review Board, and the study protocol adhered to the guidelines of the Declaration of Helsinki. Data were collected by retrospective review of the patients’ medical charts and recorded using electronic case report forms.

### 2.1. Study Population

Patients with DE accompanied by NOP features, who underwent evaluation between January 2018 and February 2020, were included in the analysis. DE was diagnosed at the first visit, based on an ocular surface disease index (OSDI) score ≥ 13 and tear break-up time (TBUT) ≤ 10 s. The inclusion criteria were as follows: (1) chronic ocular pain lasting for more than 3 months, which was unresponsive to topical lubricants (e.g., sodium hyaluronate, carboxymethylcellulose sodium, carbomer, lanolin ointment, etc.); (2) discordance between the painful DE symptoms and signs; (3) specific descriptors, including spontaneous burning, stinging, photosensitivity, and allodynia; and (4) Wong–Baker FACES Pain Rating Scale (WBFPS) score ≥ 6. The exclusion criteria were as follows: (1) topical anti-inflammatory drug use, including steroids and cyclosporin; (2) use of systemic medications that alter the pain and mood status, including analgesics, antidepressants, and antiepileptics; (3) systemic comorbidities that contraindicate the use of gabapentin, including chronic kidney disease; and (4) follow-up duration of less than 2 months.

### 2.2. Measurement of Clinical Parameters

The OSDI score, TBUT, Schirmer score, corneal stain score (CSS), and meibomian gland (MG) parameters were evaluated by the same investigator (K.C.Y.). Only the “worse” eye was assessed as follows: (1) eyes with severe CSS, or (2) the right eye if the CSS was the same in both eyes.

The OSDI questionnaire was used to quantify the vision-related QoL and included the following subscales: (1) ocular symptoms (OSDI symptoms); (2) vision-related activities of daily living (OSDI visual function); and (3) environmental triggers (OSDI trigger). The total OSDI score and each subscale score, which ranged from 0 to 100, were analyzed [21].

TBUT was assessed using a moistened fluorescein strip (Haag-Streit, Koeniz, Switzerland), and the time interval between the last complete blink and the first appearance of a dry spot or disruption of the tear film was recorded in seconds. This examination was performed thrice, and the mean TBUT value was used for the analysis. CSSs were obtained through a white light and cobalt blue filter, using the area-density index, scoring area and density of the superficial punctate corneal lesion and multiplying the area and density score [22]. Schirmer score was measured using a calibrated sterile strip (Color Bar Schirmer Tear Test; Eagle Vision Inc., Memphis, TN, USA) under topical anesthesia (0.5% proparacaine chloride). The sterile strips were placed at the lateral canthus away from the cornea and left for 5 min with the eyes closed. Schirmer scores were represented as the length of wetting in millimeters for 5 min.

The MG quality score was graded using a scale ranging from 0 to 3 as follows: grade 0, normal, clear oil expressed; grade 1, opaque, diffusely turbid, normal viscosity; grade 2, opaque, increased viscosity; and grade 3, inspissated (thick, toothpaste-like appearance) meibum or non-expressible glands. The MG expressibility score was graded by counting the central eight expressed MG orifices of the lower lid as follows: grade 0, all glands are expressible; grade 1, 3–4 glands are expressible; grade 2, 1–2 glands are expressible; and grade 3, no gland is expressible [23].

### 2.3. Assessment of Ocular Pain

The WBFPS was chosen to screen pain severity in patients with DE. We explained to the patients that each face represented a person who had no pain, had some pain, or had severe pain. Patients chose the face that best depicts the pain they were experiencing at that moment [24].

All patients completed the OPAS, which is a validated questionnaire for neuropathic pain that combines patient responses regarding ocular and non-ocular pain intensity, impact on QoL, aggravating factors, associated factors, and symptomatic relief [25]. The questions were divided into sections for analysis: questions 4–9 pertained to eye pain intensity (0 to 60); questions 10–11, pertained to non-eye pain (0 to 20); questions 13–19 (0–10, total score 0 to 60) assessed the QoL (reading and/or computer use; driving and/or watching TV; general activity; mood; sleep; and enjoying life/relations with other people); questions 20–21 (each score 0–1, total score 0 to 2) assessed aggravating factors (mechanical and chemical stimuli); and questions 22–25 (each score 0–1, total score 0 to 4), assessed associated factors (redness; burning; sensitivity to light; and tearing). The section on symptomatic relief was excluded, and only questions 4–25 were analyzed in this study.

### 2.4. Protocol of Treatment and Grouping

At the first visit, all patients were instructed to instill preservative-free sodium hyaluronate 0.15% (Hyaluni eye drops 0.15%^®^, Taejoon Pharmaceutical Co., Ltd., Seoul, Korea) 6 times a day, and loteprednol 0.5% (Lotemax^®^, Bausch & Lomb, Rochester, NY, USA) and cyclosporin A ophthalmic nanoemulsion 0.05% (Cyporin N^®^, Taejoon, Seoul, Korea) twice a day. After 1 month of treatment, an add-on of gabapentin 600 to 1200 mg/day (Neurontin cap^®^, Pfizer, New York, NY, USA) was determined according to the WBFPS score. The topical treatment was continued without gabapentin if the WBFPS score decreased by more than 2 points. If not, the add-on gabapentin treatment was administered for 1 month.

**Figure 1:**
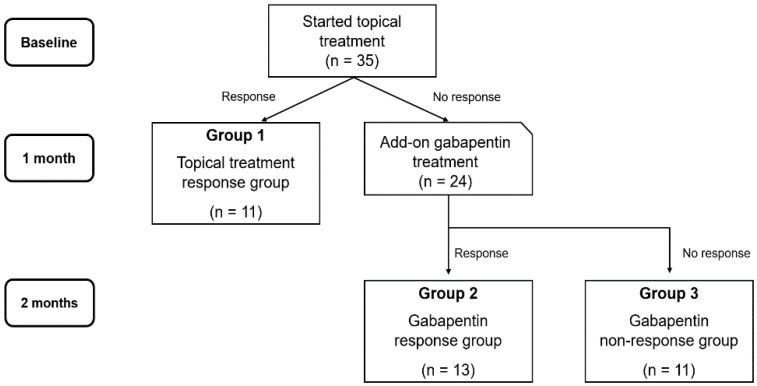
Flowchart of treatment protocol and grouping.

Patients were divided into three groups according to the treatment response: group 1 comprised patients who experienced symptomatic relief only with eye drops (topical treatment response group, *n* = 11); group 2 comprised patients who experienced symptomatic relief after the administration of gabapentin (gabapentin response group, *n* = 13); and group 3 comprised patients who were unresponsive to both treatments (gabapentin non-response group, *n* = 11; Figure 1).

### 2.5. Statistical Analysis

Statistical analyses were conducted using Statistical Package for the Social Sciences, version 22.0, for Windows (SPSS Inc., Chicago, IL, USA). The normality of distribution for all variables was assessed using the Shapiro–Wilk test. Fisher’s exact test was used for categorical data. Variables satisfying normal distribution were analyzed using the one-way analysis of variance and independent t-test, and those with non-normal distribution were analyzed with the Mann–Whitney U test. Post hoc analysis was performed after multiple comparison analysis using Tukey’s honestly significant difference test and Bonferroni adjustment. The symptom scores obtained before and after treatment were compared using the Wilcoxon signed-rank test, with differences corrected using the Benjamini–Hochberg procedure using false discovery rates of 0.25. *p*-values less than 0.05 were considered statistically significant.

## 3. Results

This study included 35 patients with DE accompanying NOP features. The mean age was 55.6 ± 11.7 years, and 27 patients (77.1%) were women. There were no differences in baseline characteristics according to sex (data not shown). Only 1 of the 24 patients treated with gabapentin experienced a side-effect (mild tremor).

Table 1 shows the demographics and medical history of the patients enrolled in this study. There was no history of systemic comorbidity, ocular surgery, and trauma in group 1. Systemic comorbidities including rheumatologic, neurologic, and phycological disorders were more frequent in group 2 than in group 3 (*p* = 0.034). No differences were observed between the previous ocular histories of groups 2 and 3.

Table 2 presents the comparisons of the clinical DE and MG parameters of the three groups. The CSS of groups 2 and 3 were lower than those of group 1 (*p* = 0.048 and *p* = 0.033). There were no differences between the CSS and other clinical parameters of groups 2 and 3.

Table 3 shows the changes in the OPAS scores of groups 1 and 2. Improved OPAS scores of ocular pain severity and associating factors were noted after treatment in group 1 (both *p* = 0.026). Group 2 exhibited improved OPAS scores of ocular pain severity, pain other than eyes, and QoL (*p* = 0.011, *p* = 0.026, and *p* = 0.011, respectively).

The comparisons of the OPAS scores between groups 2 and 3 are presented in Table 4. The scores of “enjoying life/relations with other people” associated with the QOL and pain aggravated by mechanical and chemical stimuli were lower in group 2 than in group 3 (*p* = 0.019, *p* = 0.003, and *p* = 0.004, respectively).

## 4. Discussion

Pain is an unpleasant sensory and emotional experience associated with actual or potential tissue damage, or described with respect to such damage, and can be classified into nociceptive and neuropathic pain [26]. Nociceptive pain is caused by actual or threatened damage to tissue due to the activation of nociceptors. In contrast, neuropathic pain is caused by a lesion or disease of the somatosensory nervous system. Repeated peripheral nerve injury can lead to peripheral sensitization, and prolonged peripheral ectopic pain initiates central sensitization [13,26].

DE is a multifactorial disease of the ocular surface, which is accompanied by ocular symptoms [1]. At times, patients experience ocular pain that affects their QoL. The discordance between the ocular symptoms and signs suggest an underlying neuropathic pain etiology; in such cases, ocular pain could be refractory to conventional topical DE treatment [5,8,13]. Gabapentin is the first-line treatment for systemic neuropathic pain in conditions such as fibromyalgia, postherpetic pain, and diabetic neuropathy [14]. It is an anti-convulsant drug that reduces the release of multiple excitatory neurotransmitters by acting on the α2δ subunit of the voltage-gated calcium channels, thus decreasing central sensitization [7,15].

However, limited data are available to support the use of systemic neuropathic pain medication for NOP associated with DE. A prospective, placebo-controlled study demonstrated that gabapentin reduced postoperative pain after photorefractive keratectomy [27]. However, a recent randomized pilot study showed that pregabalin, which has a similar mechanism with gabapentin, failed to prevent DE symptoms after laser-assisted in situ keratomileuses [28]. Ongun et al. [19] showed that gabapentin was more effective for the treatment of severe DE with NOP compared to topical treatment. In the present study, we aimed to analyze the differences in the clinical manifestations between groups according to treatment response in patients with DE accompanied by NOP features.

Our results showed that the frequency of other comorbidities such as rheumatologic, neurologic, and psychological disorders was higher in the gabapentin response group. Gabapentin has pharmacologic characteristics, binding to voltage-sensitive calcium channels at the α2δ subunit, affecting their function as well as influencing receptor trafficking [15]. It can secondarily influence gamma-aminobutyric acid and glutamate tone and activity via this mechanism [15]. Therefore, gabapentin can relieve not only neuropathic pain but also general systemic symptoms, such as mood, sleep, vasomotor symptoms, etc. [15,29]. This explanation corresponds to the results seen in Table 4, i.e., the significant improvement in non-ocular pain and QoL.

In contrast, the topical treatment response group had no ocular history including surgery and trauma, with more severe CSS compared to the other groups. The sensory neurons of the ocular surface and nociceptors could actually be injured in patients who had ocular surgery and trauma leading to neuroinflammation associated with peripheral and central sensitization [5,8,13]. Topical anti-inflammatory agents such as topical steroids and cyclosporin could decrease the release of proinflammatory neuropeptides and cytokines from injured nerves, affecting nociceptive pain and peripheral sensitization [9]. However, improvement of corneal nerve morphologic status and central sensitization has not been demonstrated. Therefore, patients with previous ocular history, including surgery and trauma, may not respond to topical treatment and require systemic NOP treatment.

The evoked pain in response to chemical and mechanical stimulation tended to be greater in the gabapentin non-response group than in the gabapentin response group. Evoked pain, including allodynia and hyperalgesia, is provoked or increased pain in response to stimulation [26]. These manifestations are common clinical characteristics of neuropathic pain; however, their underlying mechanisms are complex and diverse depending on the provoking stimulus [30]. The efficacy of gabapentin for the alleviation of systemic neuropathic pain was proven by several randomized, double-blind placebo-controlled studies [14]. Nevertheless, few studies have specifically focused on the treatment of evoked pain. Furthermore, one study showed that mechanical allodynia was a negative predictor of the overall effect of pregabalin in patients with postherpetic neuralgia [30,31].

Dieckmann et al. [9] suggested the proparacaine challenge test for differentiating between peripheral and central neuropathic pain and proposed a corresponding treatment strategy for DE patients with neuropathic pain etiology. The results of our study showed that the treatment response was related not only to the degree of central sensitization before treatment, but also to the patients’ systemic comorbidity, ocular history, ocular surface status, and presence of evoked pain. Gabapentin can cause side effects including CNS depression (drowsiness, dizziness, headache, etc.) and mood problems; thus, consultation with a neuropsychiatrist may be needed [15]. In this study, only one patient experienced a mild tremor as a side-effect for gabapentin treatment. Therefore, we believe gabapentin may be tolerable in DE patients associated with NOP. In addition, our results will help clinicians predict in which DE patients associated with NOP gabapentin treatment will be more effective.

Our study had some limitations. First, it was designed retrospectively and the sample size was small. Patients were recruited from a single tertiary center; thus, the findings may not be representative of the general DE population. Further prospective and longitudinal studies with a large sample size are required in the future. Second, the threshold value for determining the group classification may not have been a representative value. We classified patients with an improvement of 2 or more points on the WBFPS as the treatment response group; however, this might be not a standardized cut-off value. Moreover, we did not analyze the results of the proparacaine challenge test. It was difficult to classify the enrolled patients based on the central or peripheral phenotype of pain, since the majority of participants showed a mixed phenotype. Third, the extent of actual nerve damage was not measured using in vivo confocal microscopy. However, to the best of our knowledge, this was the first study to analyze the differences in the clinical manifestations between groups according to treatment response in DE patients with NOP features.

In conclusion, gabapentin could be successful for the treatment of DE patients with NOP features who have systemic comorbidities including rheumatological, neurological, and psychological disorders, and less evoked pain in response to mechanical and chemical stimuli. Topical treatment for DE with NOP features could be successful for patients who have a corneal staining and no ocular history including surgery and trauma.

## Figures and Tables

**Table 1:** Demographics and personal history of patients with dry eye accompanied by neuropathic ocular pain features.

	Topical Treatment (*n* = 11)	Gabapentin Treatment		*p*-Value
		Response (*n* = 13)	Non-Response (*n* = 11)	
**Demographics**				
Age (years)	59.4 ± 11.7	52.5 ± 10.3	55.5 ± 13.2	0.367
Sex (M:F)	3:8	3:10	2:9	0.879
**Comorbidities, *n***	0	7	1	0.010
Rheumatologic disease	0	3	0	
Neurologic disorder	0	2	1	
Psychological disorder	0	2	0	
**Previous ocular history, *n***	0	6	6	0.014
Cataract	0	2	1	
LASIK/LASEK	0	1	3	
Ocular trauma	0	1	1	
Eyelid surgery	0	2	1	
**Number of previously used topical agents, *n***	1.55 ± 0.69	1.77 ± 0.60	1.82 ± 0.75	0.603

M, male; F, female; LASIK, laser-assisted in situ keratomileusis; LASEK, laser-assisted sub-epithelial keratectomy. Compared using Fisher’s Exact test.

**Table 2:** Clinical parameters in patients with dry eye accompanied by neuropathic ocular pain features according to the treatment response.

	Topical Treatment (*n* = 11)	Gabapentin Treatment		*p*-Value	Post Hoc Analysis		
		Response (*n* = 13)	Non-Response (*n* = 11)		Group 1 vs. 2	Group 1 vs. 3	Group 2 vs. 3
**OSDI**	71.9 ± 7.34	69.2 ± 35.3	74.48 ± 19.1	0.959	0.993	0.994	0.956
**Tear film and ocular surface parameters**							
TBUT (sec)	4.73 ± 2.28	4.62 ± 1.94	4.00 ± 1.27	0.621	0.988	0.640	0.707
Schirmer test score (mm)	6.73± 5.31	5.85 ± 1.41	7.91 ± 2.98	0.369	0.814	0.712	0.336
CSS (0–9)	1.09 ± 1.04	0.31 ± 0.75	0.27 ± 0.47	0.031	0.048	0.033	0.994
**MG** **parameters**							
MG quality (0–3)	0.91 ± 0.83	0.85 ± 0.69	0.82 ± 0.75	0.959	0.977	0.957	0.996
MG expressibility (0–3)	0.55 ± 0.69	0.77 ± 0.60	0.69 ± 0.79	0.712	0.711	0.811	0.988

All values are presented as mean ± SD. OSDI, ocular surface disease index; TBUT, tear break-up time; CSS, corneal staining score; MG, meibomian gland. Comparison using one-way analysis of variance, and post hoc analysis using Tukey’s honestly significant difference test with Bonferroni adjustment.

**Table 3:** Changes in the Ocular Pain Assessment Survey score in the topical treatment and gabapentin response groups.

	Pre	Post	*p*-Value
**Topical treatment response group**			
Eye pain intensity (0–60)	41.0 (22.0–60.0)	29.5 (21.0–38.0)	0.026
Non–eye pain (0–20)	6.0 (5.0–7.0)	13.5 (9.0–18.0)	0.063
Quality of life (0–60)	37.5 (14.0–60.0)	34.8 (23.0–46.6)	0.113
Aggravating factors (0–2)	1.30 (1.00–1.60)	1.35 (0.80–1.90)	0.459
Associated factors (0–4)	2.85 (2.50–3.20)	2.20 (1.60–2.80)	0.026
**Gabapentin response group**			
Eye pain intensity (0–60)	38.0 (22.0–38.0)	26.0 (21.0–35.0)	0.011
Non–eye pain (0–20)	13.0 (10.0–19.0)	8.0 (6.0–16.0)	0.026
Quality of life (0–60)	43.0 (31.0–53.0)	20.0 (12.0–47.0)	0.011
Aggravating factors (0–2)	0.60 (0.20–1.40)	0.90 (0.80–1.30)	1.000
Associated factors (0–4)	1.50 (1.40–2.60)	0.80 (0.30–3.20)	0.122

All values are presented as median (interquartile range). Comparing using Wilcoxon signed-rank test.

**Table 4:** Comparison of the Ocular Pain Assessment Survey score between the gabapentin response and non-response groups.

	Gabapentin Treatment		*p*-Value
	Response (*n* = 13)	Non-Response (*n* = 11)	
**Eye pain intensity (0–60)**	39.0 (15.0–40.0)	35.0 (19.5–44.5)	0.722 ^†^
**Non–eye pain (0–20)**	8.0 (0.0–10.0)	4.0 (0.75–11.75)	0.249 ^†^
**Quality of life**			
Reading and/or computer use (0–10)	0.0 (0.0–8.0)	7.0 (4.25–9.75)	0.254 ^†^
Driving and/or watching TV (0–10)	6.67 ± 1.97	6.20 ± 2.94	0.674 *
General activity (walking, etc.) (0–10)	5.00 ± 3.30	5.40 ± 3.57	0.788 *
Mood (0–10)	1.0 (0.0–7.0)	6.5 (3.25–9.0)	0.456 ^†^
Sleep (0–10)	5.0 (5.0–6.0)	5.0 (0.0–10.0)	0.381 ^†^
Enjoying life/relations with other people (0–10)	4.67 ± 3.06	7.60 ± 2.17	0.019 *
**Aggravating factors**			
Mechanical stimuli (0–1)	0.42 ± 0.24	0.78 ± 0.28	0.003 *
Chemical stimuli (0–1)	0.40 ± 0.28	0.76 ± 0.22	0.004 *
**Associated factors**			
Redness (0–1)	0.50 ± 0.35	0.45 ± 0.32	0.628 *
Burning sensation (0–1)	0.50 ± 0.32	0.45 ± 0.42	0.381 *
Sensitivity to light (0–1)	0.60 ± 0.17	0.45 ± 0.33	0.080 *
Tearing (0–1)	0.4 (0.4–0.5)	0.3 (0.03–0.88)	0.123 ^†^

* Compared using independent t-test. ^†^ Compared using Mann–Whitney U test.
